# Characterization of a ranavirus inhibitor of the antiviral protein kinase PKR

**DOI:** 10.1186/1471-2180-11-56

**Published:** 2011-03-18

**Authors:** Stefan Rothenburg, V Gregory Chinchar, Thomas E Dever

**Affiliations:** 1 Laboratory of Gene Regulation and Development, NICHD, National Institutes of Health, Bethesda, MD 20892, USA; 2 Division of Biology, Kansas State University, Manhattan, KS 66506, USA; 3 Department of Microbiology, University of Mississippi Medical Center, Jackson, MS 39216, USA

## Abstract

**Background:**

Ranaviruses (family *Iridoviridae*) are important pathogens of lower vertebrates. However, little is known about how they circumvent the immune response of their hosts. Many ranaviruses contain a predicted protein, designated vIF2α, which shows homology with the eukaryotic translation initiation factor 2α. In analogy to distantly related proteins found in poxviruses vIF2α might act as an inhibitor of the antiviral protein kinase PKR.

**Results:**

We have characterized the function of vIF2α from *Rana catesbeiana *virus Z (RCV-Z). Multiple sequence alignments and secondary structure prediction revealed homology of vIF2α with eIF2α throughout the S1-, helical- and C-terminal domains. Genetic and biochemical analyses showed that vIF2α blocked the toxic effects of human and zebrafish PKR in a heterologous yeast system. Rather than complementing eIF2α function, vIF2α acted in a manner comparable to the vaccinia virus (VACV) K3L protein (K3), a pseudosubstrate inhibitor of PKR. Both vIF2α and K3 inhibited human PKR-mediated eIF2α phosphorylation, but not PKR autophosphorylation on Thr446. In contrast the E3L protein (E3), another poxvirus inhibitor of PKR, inhibited both Thr446 and eIF2α Ser51 phosphorylation. Interestingly, phosphorylation of eIF2α by zebrafish PKR was inhibited by vIF2α and E3, but not by K3. Effective inhibition of PKR activity coincided with increased PKR expression levels, indicative of relieved autoinhibition of PKR expression. Experiments with vIF2α deletion constructs, showed that both the N-terminal and helical domains were sufficient for inhibition of PKR, whereas the C-terminal domain was dispensable.

**Conclusions:**

Our results show that RCV-Z vIF2α is a functional inhibitor of human and zebrafish PKR, and probably functions in similar fashion as VACV K3. This constitutes an important step in understanding the interaction of ranaviruses and the host innate immune system.

## Background

Infectious diseases have devastating ecological and economical impacts on fish, amphibian and reptile populations worldwide (reviewed in [1]). Despite those effects, the precise pathogenesis of infectious diseases of ectotherm vertebrates and the interaction with the immune system of their respective hosts are mostly poorly understood. Recently, marked progress has been made in the characterization of the immune system of lower vertebrates. This has been facilitated by concentrated focus on the cloning of pathogen-induced genes and by accumulating sequence data from genome and expressed sequence tag (EST) projects. Similarly, increased information about the genomes of pathogens of lower vertebrates is becoming available. However, there are still large gaps in our knowledge, especially concerning the interaction of ectothermic pathogens with the host immune system.

Ranaviruses, which constitute a genus within the family *Iridoviridae*, are important pathogens of ectotherms and have been associated with massive die-offs of both wild and farmed populations of fish, frogs and salamanders in diverse areas of the world [2-5]. Ranaviruses are double-stranded DNA viruses with genomes ranging from 105 to 140 kb. Currently the genomes of seven ranaviruses have been sequenced: *Ambystoma tigrinum *virus (ATV, accession no. NC_005832[6]); Frog virus 3 (FV3, accession no. NC_005946[7]); Tiger frog virus (TFV, accession no. AF389451 [8]); Grouper iridovirus (GIV,accession no. AY666015 [9]; Singapore grouper iridovirus (SGIV, accession no. NC_006549[10]); Soft-shelled turtle iridovirus (STIV, accession no. EU627010 [11]); and Epizootic hematopoietic necrosis virus (EHNV, accession no. FJ433873 [12]). Phylogenetic analysis showed the existence of two major clades among ranaviruses, one that included GIV and SGIV, and another comprised of ATV, EHNV, FV3, STIV and TFV. Interestingly, the latter clade could be further subdivided with ATV and EHNV in one subclade, and FV3, STIV and TFV in the other. The diversity of organisms (amphibians, fish and reptiles) infected by viruses from this second clade, combined with short branch lengths within its two subclades may indicate recent host switches among ranaviruses [12]. However, it should be noted that the host range of ranaviruses is incompletely understood at this time.

The host immune system has evolved multiple ways to fight virus infection and replication. One important arm of the host immune response is the innate immune system, which recognizes molecular patterns present in many pathogens and initiates antimicrobial responses [13,14]. An important component of the host response is the antiviral protein kinase PKR, which contains double-stranded (ds) RNA binding domains (RBD) and a kinase domain. PKR is activated by dsRNA, which is formed during infection by many RNA and DNA viruses, and phosphorylates the α subunit of eukaryotic translation initiation factor 2 (reviewed in [15]). PKR is inactive in its latent monomeric form. However, upon binding dsRNA, two PKR molecules dimerize and undergo autophosphorylation on residue Thr446 (for human PKR) [16-18]. Activated PKR then phosphorylates eIF2α on Ser51, which subsequently acts as an inhibitor of the guanine nucleotide exchange factor eIF2B. As eIF2B normally exchanges GDP for GTP on eIF2, a step necessary for successful translation initiation, eIF2α phosphorylation leads to a general inhibition of translation initiation [19,20]. The function of mammalian PKR and its interaction with viruses has been extensively characterized (reviewed in [15]). However, PKR-like molecules in ectotherms eluded molecular characterization until recently. PKR-like activity was first described in fish cells [21,22]. This was followed by the cloning and functional characterization of crucian carp and zebrafish PKR-related genes, which contain Z-DNA binding (Zα) domains instead of the dsRBDs and were hence named PKZ [23,24]. PKZ was subsequently described in Atlantic salmon and the rare minnow [25,26]. Recently, authentic PKR genes were described and characterized in many ectotherm species including zebrafish, pufferfish, Japanese flounder and two *Xenopus *species [27,28]. Like mammalian PKR, both PKZ and PKR are induced by immunostimulation [23,27,28]. Phylogenetic analyses indicate that a duplication of an ancestral PKR-like gene in the fish lineage probably led to the emergence of PKR and PKZ in a fish ancestor, and might have helped to extend the spectrum of viral nucleic acids that can be recognized [27]. Although higher vertebrates lack PKZ genes, they contain a different Zα-containing protein, termed ZBP1, which binds Z-DNA and has been implicated in the recognition of viral DNA and the induction of an antiviral response [29-31].

In order to overcome the antiviral effects of PKR many mammalian viruses encode inhibitors of PKR, which block PKR activation or activity at different steps during or following the activation process (reviewed in [32]). Many poxviruses possess two PKR inhibitors, which are designated E3 and K3 in vaccinia virus (VACV) and are encoded by the E3L and K3L genes, respectively. E3 binds to dsRNA and prevents activation of PKR [33,34], whereas K3 encodes an S1 domain that is homologous to the N-terminus of eIF2α and inhibits activated PKR by binding to the kinase domain and acting as a pseudosubstrate inhibitor of PKR [18,35,36].

Interestingly, most ranaviruses encode a protein with an S1 domain, which is related to the S1 domain of eIF2α and K3 and is referred to as a viral homolog of eIF2α or vIF2α. In contrast to K3, which only possesses the S1 domain, vIF2α proteins contain a C-terminal extension of between 165 to 190 amino acids, for which no sequence homology to any other proteins was described. It was previously speculated that vIF2α in analogy to K3 might be an inhibitor of PKR and might therefore play an important role in the pathogenesis of ranaviruses [37-39]. Herein, using a heterologous yeast assay system, we describe the characterization of vIF2α as an inhibitor of human and zebrafish PKR.

## Results

**Figure 1:**
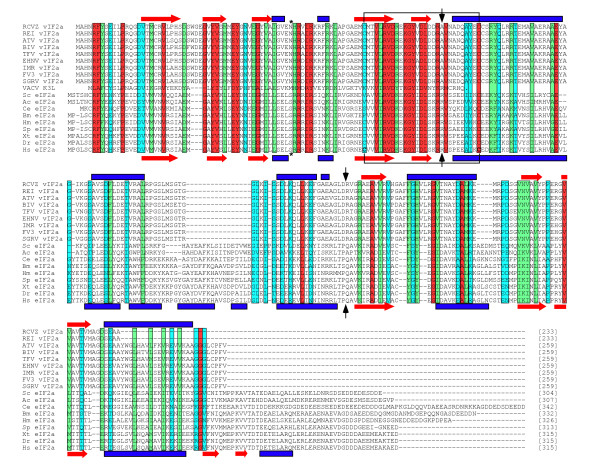
**Multiple sequence alignment of vIF2α and eIF2α sequences**. Multiple sequence alignment with the indicated sequences was generated using MUSCLE [54]. The background of residues that are highly conserved between vIF2α and eIF2α are colored as follows: 100% identity: red; identical or conservative substitutions: green; residues that are 100% conserved in all vIF2α sequences and found in some eIF2α sequences: light blue. Secondary structure elements as reported for human eIF2α [41] are shown below the sequences: β-strand: red arrow; α-helix: blue box. Vertical arrows indicate boundaries between S1, helical, and C-terminal domains in eIF2α. Secondary structure elements that were predicted for RCV-Z and ATV vIF2α using Porter are shown above the alignments [55]. Cysteines that form a disulfide bridge in the crystal structure of human eIF2α are shown in bold and connected by lines. An asterisk marks the position of Ser 51, which is phosphorylated in eIF2α. Species abbreviations and sequence accession numbers are as follows: RCVZ *= Rana catesbeiana *virus Z, AAY86037; REI = *Rana esculenta *iridovirus, AAG43853; EHNV = *Epizootic haematopoietic necrosis *virus, CAB37351; TFV = Tiger frog virus, AAL77798; BIV = *Bohle iridovirus*; ABN50368; FV3 = Frog virus 3, AAD38359; SGRV = *Silurus glanis *ranavirus, AAD38355; ATV = *Ambystoma tigrinum *virus, YP_003830; IMR = *Ictalurus melas *ranavirus, AAD38356; VACV = Vaccinia virus WR, YP_232916; Hs = *Homo sapiens*, NP_004085; Xt = *Xenopus tropicalis*, NP_001005630; Dr= *Danio rerio*, NP_955863; Sp = *Strongylocentrotus purpuratus*, XP_779939; Hm = *Hydra magnipapillata*; XP_002156465; Bm = *Bombyx mori*, NP_001037516; Ce = *Caenorhabditis elegans*, NP_490930; Sc = *SaccharoMyces cerevisiae*, NP_012540; Ac = *Aspergillus clavatus*, XP_001271371.

We present three lines of evidence that the C-terminus of vIF2α is actually homologous to the helical and parts of the C-terminal domains of eIF2α. Firstly, we performed PSI-BLAST searches with vIF2α from ATV and RCV-Z. During the first iteration, sequence similarity for regions spanning amino acids 5-118 of ATV-vIF2α with the S1 and helical domains eIF2α from multiple eukaryotes was noted. During the second iteration, this region of similarity to eIF2α was extended to amino acid position 253 of vIF2α. Secondly, multiple sequence alignments including vIF2α from many ranaviruses and eIF2α from a diverse set of eukaryotes showed conservation of amino acids outside the S1 domain: 8 amino acids are 100% conserved among the sequences (Figure 1, red background; Cys99, Glu118, Leu160, Ala177, Gly192, Ala199, Val220 and Gly253). Moreover, conservative amino acid differences are present at 22 positions outside the S1 domain (Figure 1, green background). At many other positions, amino acids that are identical to the ones found in vIF2α are present in a subset of eIF2α sequences (Figure 1, light blue background). While the multiple sequence alignment reveals sequence homology between vIF2α and eIF2α throughout the reading frame, sequence similarity is highest within the S1 domain, with the highest levels of sequence identity surrounding strands β4 and β5 (Val74 - Leu88 in vIF2α) as previously described [38,39]. Interestingly, in VACV K3 this region was previously shown to be important for PKR inhibition [40]. Thirdly, secondary structure prediction with ATV and RCV-Z vIF2α resulted in predicted β-sheets and α-helices that coincide very well with the solved structural features observed in the NMR structure of human eIF2α [41]. These observations indicate that the middle and C-terminal parts of vIF2α are homologous to the helical and C-terminal domains, respectively, of eIF2α.

**Figure 2:**
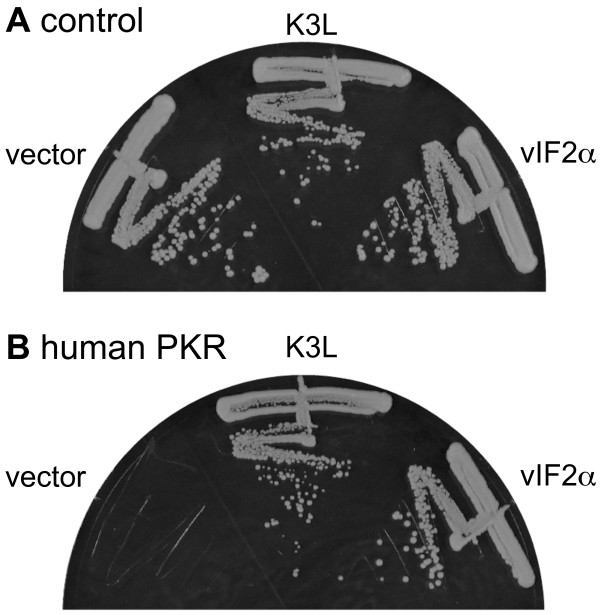
**vIF2α inhibits human PKR-mediated toxicity in yeast**. Plasmids expressing VACV K3L (pC140) or RCV-Z vIF2α (pC3853) under the control of a yeast *GAL-CYC1 *hybrid promoter, or the vector pEMBLyex4 alone, were introduced into isogenic yeast strains having either an empty vector (A, control, J673) or a *GAL-CYC1*-human PKR construct (B, J983) integrated at the *LEU2 *locus. The indicated transformants were streaked on SC-Gal medium where expression of both PKR and the viral proteins was induced, and incubated at 30°C for 4 days. Results shown are representative of 4 independent transformants for each plasmid.

Yeast-based assays were previously employed to characterize PKR and its interaction with viral inhibitors [34,40,42,43]. To test whether vIF2α can inhibit PKR-mediated toxicity in yeast, we transformed a control strain and a strain expressing human PKR under the control of the galactose-regulated *GAL-CYC1 *hybrid promoter with plasmids designed to express RCV-Z vIF2α and VACV K3L also under control of the *GAL-CYC1 *promoter. When grown under inducing conditions (galactose medium), comparable growth was seen in the control strain transformed with vector, K3L or vIF2α, demonstrating that K3 and vIF2α had no effect on yeast cell growth (Figure 2A). In contrast, induction of PKR expression was toxic in the vector-transformed yeast, whereas the toxicity was suppressed by co-expression of K3L or vIF2α (Figure 2B).

**Figure 3:**
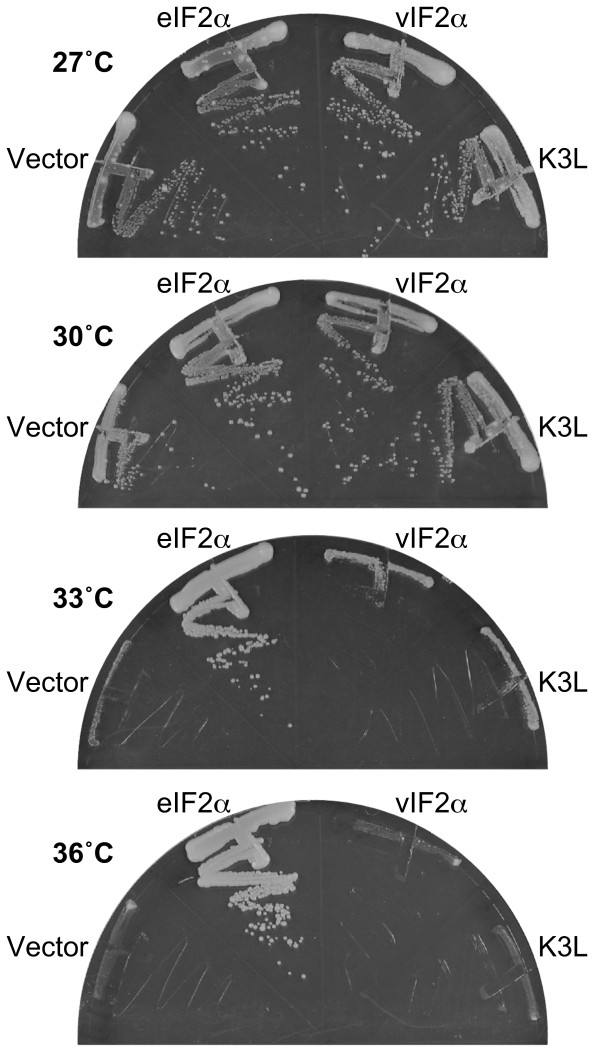
**vIF2α does not complement eIF2α function in yeast**. Plasmids expressing VACV K3L (pC140) or RCV-Z vIF2α (pC3853) under the control of a yeast *GAL-CYC1 *hybrid promoter, or yeast eIF2α (p919) under the control of its native promoter, or the vector pEMBLyex4, were introduced into the temperature-sensitive eIF2α (*sui2-1*, TD304-10B) mutant strain. The indicated transformants were streaked on SC-Gal medium, where eIF2α expression was maintained and the viral protein expression was induced, and incubated at the indicated temperatures. Results shown are representative of 4 independent transformants for each plasmid.

Based on the homology of vIF2α with eIF2α throughout the entire ORF we tested whether suppression of PKR toxicity might be caused by the complementation of eIF2α function by vIF2α. To this end, we transformed a yeast strain that carries a temperature-sensitive mutant of eIF2α (*sui2-1*) [44] with an empty vector, with a plasmid designed to express wild-type eIF2α (*SUI2*) under the control of its native promoter, or with the plasmids that express vIF2α or K3L under the control of the galactose regulated *GAL-CYC1 *promoter. Yeast transformants were streaked on synthetic complete medium containing galactose (SC-Gal) and incubated at different temperatures. At permissive temperatures (27°C and 30°C) all transformants grew well (Figure 3). However, when incubated at restrictive temperatures (33°C and 36°C), only wild type eIF2α was able to rescue growth (Figure 3). It is important to note that under these growth conditions vIF2α and K3L were able to suppress PKR toxicity (data not shown), indicating that the viral proteins are functional under these conditions. As expression of neither vIF2α nor K3L suppressed the growth defects of the *sui2-1 *mutant strain, we conclude that vIF2α does not functionally substitute for eIF2α.

**Figure 4:**
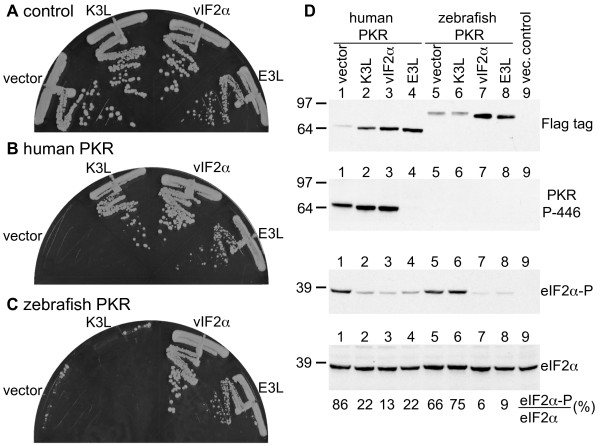
**Sensitivity of human and zebrafish PKR to inhibition by vIF2α K3 and E3**. Plasmids expressing VACV K3L (pC140), RCV-Z vIF2α (pC3853), or VACV E3L (p2245) under the control of a yeast *GAL-CYC1 *hybrid promoter, or the empty vector pEMBLyex4, were introduced into isogenic yeast strains having either an empty vector (A, J673), a *GAL-CYC1*-human PKR construct (B, J983), or a *GAL-CYC1*-zebrafish PKR construct (C, J944) integrated at the *LEU2 *locus. The indicated transformants were streaked on SC-Gal medium where expression of both PKR and the viral proteins was induced, and incubated at 30°C for 4 days. Results shown are representative of 4 independent transformants for each plasmid. (D) Transformants described in panels A-C were grown in liquid SC-Gal medium for 13 hours, then whole cell extracts were obtained from equal numbers of cells and subjected to SDS-PAGE followed by immunoblot analysis. Following transfer to nitrocellulose membranes, the upper halves of the blots were probed with phosphospecific antibodies against Thr446 in human PKR (second panel from top), then stripped and probed with anti-Flag tag antibodies which detect Flag-tagged human and zebrafish PKR (top panel). The lower part of the blot was incubated with phosphospecific antibodies against Ser51 in eIF2α (eIF2α-P; third panel from top), then stripped and probed with polyclonal antiserum against total yeast eIF2α. Lane 9 contains protein extracts from the vector (pEMBLyex4) transformed control strain (J673, panel A). The ratios between phosphorylated eIF2α and total eIF2α converted to percentages are shown below.

We next compared the effect of vIF2α on human and zebrafish PKR with the effects of the two VACV PKR inhibitors K3 and E3. In the control strain not expressing PKR, expression of K3L or vIF2α had no effect on yeast cell growth, whereas expression of E3L induced a slow growth phenotype as previously described [34] (Figure 4A). The toxicity associated with expression of human PKR was inhibited by co-expression of K3L, vIF2α or E3L (Figure 4B). Interestingly, the toxicity associated with expression of zebrafish PKR in yeast was only inhibited by vIF2α or E3L, but not by K3L (Figure 4C). Thus in accord with the virus host range vIF2α, but not VACV K3L, may have evolved to inhibit fish PKR. To assess the effectiveness of K3, E3, and vIF2α to inhibit human and zebrafish PKR, matching sets of strains expressing a particular inhibitor and either no PKR, human PKR, or zebrafish PKR were streaked on the same plate for comparison. Examining the colony sizes of the transformants in the streaks revealed that K3 did not fully inhibit human PKR (colonies of cells expressing human PKR plus K3L were smaller than colonies expressing K3L and no PKR, Additional file 1: Figure S1A). In contrast, vIF2α and E3 appeared to fully inhibit both human and zebrafish PKR (Additional file 1: Figure S1B, C).

Suppression of PKR toxicity in yeast could be due to impaired PKR expression or due to inhibition of eIF2α phosphorylation. In order to examine eIF2α phosphorylation, yeast whole cell extracts were prepared by the TCA method to prevent protein degradation and dephosphorylation, and Western blot analyses were performed using phospho-specific antibodies directed against phospho-Ser51 in eIF2α. To normalize for protein loading, the blot was then stripped and probed with anti-yeast eIF2α antiserum. As shown in Figure 4D (next to bottom panel), induction of either human or zebrafish PKR expression in the absence of a viral inhibitor led to high levels of eIF2α phosphorylation. Co-expression of K3L, vIF2α, or E3L greatly reduced eIF2α phosphorylation in cells expressing human PKR (Figure 4D and Additional file 2: Figure S2). Consistent with the growth assays, vIF2α and E3, but not K3, inhibited eIF2α phosphorylation in yeast expressing zebrafish PKR. Next, PKR expression levels were monitored using an anti-Flag tag antibody. Expression levels of PKR were higher in the presence of effective eIF2α phosphorylation inhibitors. As observed previously PKR autoinhibits its own expression in yeast [34,40,45]. Presumably PKR phosphorylation of eIF2α leads to suppression of total protein synthesis including PKR expression. Accordingly, inhibition of PKR by the viral inhibitors restores protein synthesis and leads to higher PKR levels. Taken together, the results of the PKR expression and eIF2α phosphorylation studies demonstrate that vIF2α can effectively inhibit eIF2α phosphorylation by human and zebrafish PKR.

In the presence of effective eIF2α phosphorylation inhibitors, PKR migrated faster on SDS-PAGE than in the controls (Figure 4D, top panel, lanes 2-4 versus 1 and lanes 7-8 versus 5). This might have been caused by inhibition of PKR autophosphorylation. To examine PKR autophosphorylation, we probed the Western blots with a phospho-specific antibody that recognizes human PKR phosphorylated on Thr446. High levels of Thr446 phosphorylation were detected in the absence of inhibitors and when either K3 or vIF2α were present. Thr446 phosphorylation was effectively inhibited in the presence of E3 (Figure 4D, second panel, lanes 1-4). These results indicate that K3 and vIF2α are unable to block Thr446 phosphorylation and are consistent with previous findings that K3 binding to PKR is dependent on Thr446 phosphorylation [18]. Presumably vIF2α, like K3, binds to PKR following autophosphorylation on Thr446 and blocks subsequent autophosphorylation events that lead to altered mobility of PKR on SDS-PAGE. Zebrafish PKR was not detected with the antibody directed against Thr446-phosphorylated human PKR (Figure 4D, second panel, lanes 5-8). This was expected because of the strong sequence divergence between human and zebrafish PKR surrounding the phosphorylation site [27]. Finally, using yeast growth rate assays as described above, vIF2α was found to inhibit, at least partially, both *Xenopus laevis *PKR1 and zebrafish PKZ (data not shown). However, precise determination of PKR1 and PKZ sensitivity to vIF2α inhibition will depend on the ability to obtain yeast strains expressing the appropriate level of each kinase.

**Figure 5:**
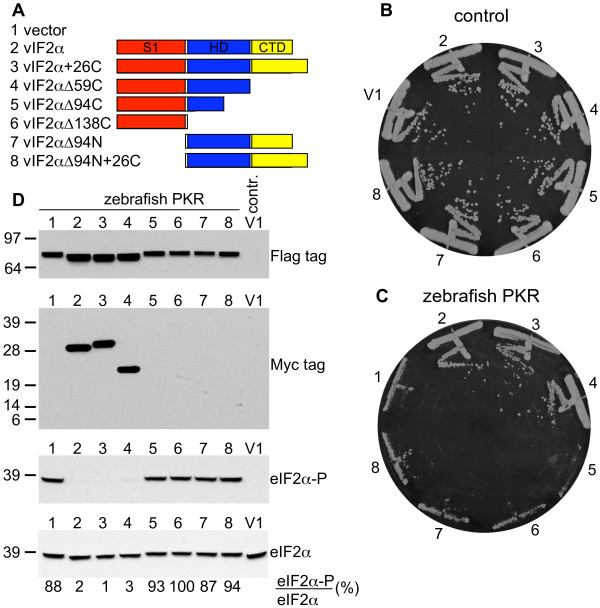
**Both S1 and helical domains in vIF2α are required for PKR inhibition**. (A) Schematic representation of RCV-Z vIF2α constructs tested in yeast growth assays and Western blots analyses. S1 domain (red), helical domain (HD; blue) and C-terminal domain (CTD, yellow) are represented by boxes. Numbers that follow deltas (Δ) indicate the(number of residues that were deleted from the C- or N-terminus, respectively. The extended C-terminus (26 amino acids) from ATV vIF2α was added to the C-terminus of RCV-Z vIF2α in the constructs with the +26C label. The indicated constructs were introduced into isogenic yeast strains having either an empty vector (B, J673) or a *GAL-CYC1*-zebrafish PKR construct (C, J944) integrated at the *LEU2 *locus. The indicated transformants were streaked on SC-Gal medium where expression of both PKR and the viral proteins was induced, and incubated at 30°C for 4 days. Results shown are representative of 4 independent transformants for each plasmid. (D) Transformants described in panels B-C were grown in liquid SC-Gal medium for 13 hours, then whole cell extracts were obtained from equal numbers of cells and subjected to SDS-PAGE followed by immunoblot analysis. Following transfer to nitrocellulose membranes, the upper half of the blot was probed with anti-Flag tag antibodies, which detect Flag-tagged zebrafish PKR (top panel). The lower part of the blot was incubated with anti-Myc tag antibodies to detect Myc-tagged vIF2α (second panel from top), then stripped and probed with phosphospecific antibodies against Ser51 in eIF2α (eIF2α-P; third panel from top), and finally stripped again and probed with polyclonal antiserum against total yeast eIF2α (bottom panel). The ratios between phosphorylated eIF2α and total eIF2α converted to percentages are shown below.

In order to test which domains of vIF2α are important for PKR inhibition we tested various vIF2α deletion mutants for their ability to inhibit PKR activity. Additionally, the C-terminus of RCV-Z vIF2α was extended to match the length of ATV vIF2α (see Figure 1). For the latter constructs, the 26 C-terminal amino acids found in ATV vIF2α that are not in RCV-Z vIF2α due to an early termination codon were appended to the C-terminus of RCV-Z vIF2α (vIF2α+26C, Figure 5A). None of the vIF2α constructs led to a growth defect in the control strain not expressing PKR (Figure 5B). In a zebrafish PKR-expressing strain, wild-type vIF2α, vIF2α+26C, and vIF2αΔ59C (lacking the C-terminal 59 amino acids) led to comparable inhibition of PKR toxicity (Figure 5C, sectors 2-4 versus 1). In contrast, no PKR suppression was observed when the helical domain was partly (vIF2αΔ94C) or completely (vIF2 αΔ138C) deleted or when the N-terminus was deleted (vIF2αΔ94N and vIF2 αΔ94N+26C) (Figure 5C, sectors 5-8). Western analyses of eIF2α phosphorylation in the strains expressing zebrafish PKR and the various vIF2α mutants revealed that vIF2α, vIF2α+26C, vIF2α59C led to strong and comparable inhibition of eIF2α phosphorylation (Figure 5D, next to bottom panel, lanes 2-4). Consistent with their inability to inhibit PKR toxicity in yeast, high levels of eIF2α phosphorylation were observed in strains expressing the other vIF2α mutants (Figure 5D). As seen earlier, PKR was expressed at higher levels and migrated faster on SDS-PAGE when PKR toxicity and eIF2α phosphorylation were suppressed (Figure 5D, top panel). Western blot analyses using antibodies against a C-terminal Myc-epitope tag in the vIF2α constructs revealed detectable expression for only vIF2α, vIF2α+26C, and vIF2α59C. Comparable results were obtained in Western blot analyses of protein extracts from the control (-PKR) strain expressing these same vIF2α mutants (data not shown), indicating that both the S1 domain and the helical domain are essential for vIF2α expression and/or stability.

## Discussion

Ranaviruses are important pathogens of fish, amphibians and reptiles (reviewed in [2]). However, little is known about how they interact with the immune system of their hosts. Herein we show that RCV-Z vIF2α, a homolog of eIF2α, is an effective inhibitor of PKR in a heterologous yeast assay system. PKR is an important antiviral protein kinase that has been primarily studied in mammals (reviewed in [15]). PKR-related genes have recently been identified in a variety of fish and amphibian species. Fish PKR genes are expressed at low levels constitutively, but they are highly induced after viral infection and stimulation with the dsRNA analog poly(I:C), which mimics viral infection [27,28]. It was recently shown that PKR of the Japanese flounder (*Paralichthys olivaceus*) was able to inhibit replication of *Scophthalmus maximus rhabdovirus *[28]. To date, only PKR inhibitors from mammalian viruses have been functionally characterized (reviewed in [32]). Moreover, the only well-characterized viral PKR inhibitors that directly target the PKR kinase domain are the pseudosubstrates found in many poxviruses and represented by VACV K3L, which is homologous to the S1 domain of the PKR target eIF2α [33,40,46,47]. It was speculated that the ranavirus vIF2α protein, another eIF2α homolog, might inhibit PKR of infected hosts [38,39]. A notable difference between K3 and eIF2α is the presence of an extended C-terminal domain in eIF2α. In addition to the C-terminal α/β domain, eIF2α consists of an N-terminal S1 domain and a central α-helical domain. The K3 protein is homologous to the N-terminal domain in eIF2α. Like K3, vIF2α shows moderate sequence identity to eIF2α in the S1 domain. In this study we used PSI-BLAST analyses, multiple sequence alignment and secondary structure prediction to show that the C-terminal parts of vIF2α are likewise homologous to the helical and C-terminal domains of eIF2α.

Functional analyses using deletion constructs of vIF2α revealed that both the S1 and helical domains are sufficient for inhibition of PKR in yeast (Figure 5). Since the presence of both domains was necessary for detectable vIF2α expression, it appears possible that the domains are important to stabilize each other. The crystal structure of human eIF2α showed that the S1 and helical domains are connected by an intramolecular disulfide bridge formed by cysteine residues 69 and 97 [48]. Interestingly, a cysteine corresponding to position 69 is found in many Metazoa, including Chordata, Echinodermata, Cnidaria and Mollusca, but is missing in most Arthropoda (except *Ioxedes scapularis*), in all fungi and plants sequences currently found in Genbank, and in all poxviral K3L orthologs (Figure 1 and data not shown). As cysteines corresponding to residues 69 and 97 in human eIF2α are found in all vIF2α sequences (Figure 1), we speculate that formation of a disulfide bridge between the two domains might be important for the stability of vIF2α. Future *in vitro *kinase assays with vIF2α constructs that are produced in a cell-free translation system might be suited to further investigate the importance of the individual domains.

It is striking that eIF2α sequences and all known vIF2α sequences display a high level of sequence identity within their respective groups. The sequence identity for eIF2α is between 92% and 100% among vertebrates, while the sequence identity for vIF2α is between 95% and 98% among ranaviruses. In contrast, K3L orthologs are very diverse, some of which display only around 30-40% sequence identity to each other [49]. The high sequence conservation in eIF2α and vIF2α indicates that eIF2α might be under purifying (negative) selective pressure in order to maintain its primary sequence or, alternatively, that current ranaviruses might have experienced bottlenecks in their recent evolution. Overall the S1 domains of vIF2α and K3 are comparably distantly related to eIF2α.

Interestingly, some Ranaviruses do not encode functional vIF2α orthologs. GIV and SGIV do not contain vIF2α orthologs, and truncated vIF2α genes lacking regions of the N-terminal and the helical domains are found in the completely sequenced FV3 strain and in STIV [7,11]. As our studies indicate that the N-terminus of vIF2α is essential for PKR inhibition, these complete or partial deletions might lead to the attenuation of the viruses. In accord with this notion FV3, which lacks most of vIF2α, is much less pathogenic than RCV-Z in North American bullfrog (Rana catesbeiana) tadpoles [39]. Alternatively the absence of predicted functional vIF2α proteins in some ranaviruses suggests that, as in vaccinia virus, a second PKR inhibitor may be present in ranaviruses.

Western blot analyses showed that human PKR was expressed at higher levels in yeast expressing the PKR inhibitors vIF2α, K3L, or E3L, consistent with the notion that the viral inhibitors suppress autoinhibition of PKR expression. Moreover, PKR from cells expressing viral inhibitors migrated faster on SDS-PAGE, suggesting that the inhibitors might block PKR autophosphorylation. Thr446 is the only site in the human PKR kinase domain that is stoichiometrically phosphorylated and visible in the PKR crystal structure, where it is thought to stabilize the active PKR conformation [18,50]. Once activated, PKR can phosphorylate eIF2α as well as autophosphorylate other residues in the kinase [17,34]; however the significance of the latter is not fully understood. Interestingly, only E3L was able to prevent Thr446 phosphorylation. In cells expressing K3L or vIF2α, Thr446 was phosphorylated to the level observed in the absence of an inhibitor, whereas PKR mobility was comparable to that in E3L transformed cells. A likely explanation is that K3 and vIF2α bind after the initial Thr446 autophosphorylation and block subsequent phosphorylation events. This is in agreement with the finding that activated WT PKR, but not the PKR-T446A mutant, was able to bind K3 [18].

Like human PKR, zebrafish PKR was inhibited by E3 and vIF2α. Moreover, as was seen for human PKR, zebrafish PKR from cells expressing the inhibitors migrated faster on SDS-PAGE, indicative of blocked secondary phosphorylation events. An interesting difference between human and zebrafish PKR is that zebrafish PKR was resistant to K3 inhibition in both the growth and eIF2α phosphorylation assays. In accord with our previous studies on PKR inhibition by K3 [49], we propose that K3L might have evolved to suppress PKR of the natural poxvirus hosts and that zebrafish PKR is too different to be targeted with high efficiency. It is not clear why vIF2α, which is found in amphibian and fish viruses, can inhibit both human and zebrafish PKR, but it is possible that vIF2α targets more conserved residues in the PKR kinase domain than does K3. Previously we showed that K3 exhibits species specificity for inhibition of PKR. Whereas human PKR was only moderately inhibited by VACV K3, mouse PKR was much more sensitive [49]. This difference in sensitivity was attributed to residues that were subject to positive selection during evolution. Interestingly, positive selection was also observed in the kinase domains of fish and amphibian PKR and fish PKZ [49]. It will be interesting to determine whether vIF2α also shows altered specificity for PKR or the related PKZ of the species that are naturally infected with vIF2α-containing ranaviruses.

## Conclusions

Overall, it appears that vIF2α and K3 inhibit PKR in a similar fashion, by acting as pseudosubstrates and inhibiting PKR following kinase activation. As vIF2α does not act as an eIF2α substitute, but instead inhibits PKR function, the renaming of vIF2α might be considered. We suggest changing the name from vIF2α to RIPR, the acronym for Ranavirus Inhibitor of Protein kinase R.

## Methods

### Yeast strains and plasmids

Human (hs) and zebrafish (dr) PKR cDNAs containing both N-terminal His6- and Flag tags were first cloned into the yeast expression vector pYX113 (R&D systems) under the control of a *GAL-CYC1 *hybrid promoter [27]. Next, the two DNA fragments containing the *GAL-CYC1 *promoter and a PKR cDNA were subcloned into the *LEU2 *integrating vector pRS305, which was then directed to integrate into the *leu2 *locus of the strain H2557 (*MAT*α *ura3-52 leu2-3 leu2-112 trp1Δ63 gcn2Δ*) generating the strains J983 (*MAT*α *ura3-52 leu2-3 leu2-112 trp1Δ63 gcn2Δ <GAL-CYC1-hsPKR, LEU2 >*) and J944 (*MAT*α *ura3-52 leu2-3 leu2-112 trp1Δ63 gcn2Δ <GAL-CYC1-drPKR, LEU2 >*). Construction of the control strain J673 (*MAT*α *ura3-52 leu2-3 leu2-112 trp1Δ63 gcn2Δ <LEU2 >*) was described previously [51]. The temperature-sensitive eIF2α strain TD304-10B (*MAT*α *his4-303 ura3-52 leu2-3 leu2-112 sui2-1*) is a derivative of the previously described *sui2-1 *strain 117-8AR20 [44].

A DNA fragment encoding RCV-Z vIF2α [39] was amplified by PCR using viral DNA as a template and primers C27 (5'- TAGGATCCAAAATGGCACACAACAGGTTTTAC-3') and C28 (5'- TAAAGTCGACCGCCGCCTCAGAGTCGCCGG-3'). The PCR product was then subcloned between the *Bam*H I and *Sal *I restrictions sites of a modified version of the yeast expression vector pEMBLyex4 that contains two Myc tags at the C-terminal end of the multiple cloning site (pC3852) generating the plasmid pC3853. The following primer combinations were used for cloning of vIF2α mutant constructs: vIF2αΔ59C: C27 plus C29 (5'- TAAAGTCGACCCGACCGACTCTGTCGAGGC-3'); vIF2αΔ94C: C27 plus C30 (5'-TAAAGTCGACTCTCAGGGCCCTCACGGTCTC-3'); vIF2αΔ138C: C27 plus C31 (5'-TAAAGTCGACCTGATCGGCATTCACGGC-3'); vIF2α+26C: C27 plus C32 (5'-TAAAGTCGACCACAAAGGGGCACAGTCCTC-3'); vIF2αΔ94N: C33 (5'- TAGGATCCAAAATGGCCGATCAGGCGTACGAGTG-3') plus C28; and vIF2αΔ94N+26C: C33 plus C32. The plasmid template for vIF2α+26C and vIF2αΔ94N+26C was generated by fusion PCR using vector primer C23 (5'- CATATGGCATGCATGTGCTCTG-3') plus primer C21 (5'- GCCTTTACGACCTCTCGCACCTCAGACAGCACGGCGTGCAGTCCCCAGTAC GCCGCCTCAGAGTCGCCG-3') for the first PCR and primer C22 (5'- GTGCGAGAGGTCGTAAAGGCTGCCGGGGGAGGACTGTGCCCCTTTGTGTA AGTCGACCTGCAGGCATGC-3') plus vector primer C24 (5'- CGCTTCCGAAAATGCAACGC-3') for the second PCR. Following PCR purification, the two PCR products were mixed and used as a template for PCR along with the vector primers A46F (5'-ATTCTTTCCTTATACATTAGGTCC-3') and A20R (5'-TGCTGCCACTCCTCAATTGG-3'). Finally, the PCR products were cloned into the *Bam*HI and *Sal*I sites of pEMBLyex4. All PCRs were carried out using Pfu Polymerase (Stratagene) and all plasmids were sequenced to verify correct sequences. Derivatives of pEMBLyex4 expressing VACV K3L (pC140) and VACV E3L (p2245), as well as the low copy-number *SUI2, URA3 *plasmid p919 were described previously [34,40,52].

Yeast strains were transformed using the LiAcetate/PEG transformation method. For each transformation, four independent colonies were analyzed by streaking on inducing medium, SC-Gal minus uracil (synthetic complete medium containing 2% galactose and all amino acids, but lacking uracil) and grown at 30°C if not otherwise indicated.

### Protein expression and Western Blot analyses

Yeast transformants were grown to saturation in 2 ml of SD medium. This starter culture was diluted 1:50 in 25 ml SD medium and grown to OD_600 _= 0.6 and then shifted to SC-Gal medium to induce expression. After 13 hours, ODs of the cultures were measured and carefully adjusted by dilution in water to obtain comparable ODs and thus to lyse equivalent amounts of cells for each sample. Whole-cell extracts (WCEs) were prepared using the trichloroacetic acid (TCA) method as described previously [53] and then suspended in 200 μl 1.5 × loading buffer with reducing agent (both Invitrogen) and neutralized by the addition of 100 μl 1 M Tris base. Samples (5 μl) were fractionated on 10% Bis-Tris gels (Invitrogen), run in MOPS buffer (Invitrogen), and then transferred to nitrocellulose membranes. Upper parts of the membranes were incubated with rabbit phosphospecific anti-human PKR Thr446 antibodies (21st Century Biochemicals) and then stripped and probed with anti-D (Flag) tag antibody (Applied Biological Materials). Lower halves of the membranes were incubated with an anti-Myc tag antibody (Applied Biological Materials), rabbit phosphospecific antibodies directed against phosphorylated Ser51 of eIF2α (BioSource International), or rabbit polyclonal antiserum against total yeast eIF2α Immune complexes were detected using enhanced chemiluminescence. Band intensities were quantified by densitometry using ImageJ http://rsbweb.nih.gov/ij/ and ratios between phosphorylated eIF2α and total eIF2α were calculated.

### Multiple sequence alignment and secondary structure prediction

Multiple sequence alignments of all sequences shown in Figure 1 plus all poxvirus K3L orthologs listed in [49] were performed using MUSCLE [54]. Secondary structure predictions for RCV-Z and ATV vIF2α sequences were performed using Porter [55].

## Authors' contributions

SR and TED devised this study with important input from VGC. All experiments were performed by SR. The manuscript was drafted by SR with essential contributions from TED and VGC. All authors read and approved the final manuscript.

## Supplementary Material

Additional file 1 **Figure S1 Comparison of colony sizes of PKR-expressing and control stains expressing K3L, vIF2α or E3L**. Plasmids expressing VACV K3L (A, pC140), RCV-Z vIF2α (B, pC3853), or VACV E3L (C, p2245) under the control of a yeast *GAL-CYC1 *hybrid promoter were introduced into isogenic yeast strains having either an empty vector (J673), a *GAL-CYC1*-human PKR construct (hsPKR, J983), or a *GAL-CYC1*-zebrafish PKR construct (drPKR, J944) integrated at the *LEU2 *locus. The indicated transformants were streaked on SC-Gal medium where expression of both PKR and the viral proteins was induced, and incubated at 30°C for 4 days. Results shown are representative of 4 independent transformants for each plasmid. Click here for file

Additional file 2 **Figure S2 Relative PKR-induced eIF2α phosphorylation levels after expression of vIF2α, K3L or E3L**. Using data from Figure 4D and an independent experiment, the band intensities of phosphorylated and total eIF2α obtained from Western blots of TCA extracts of yeast cells expressing either human or zebrafish PKR and transformed with an empty vector or plasmids expressing K3L, vIF2α or E3L, as indicated, were measured using ImageJ. The ratios of phosphorylated and total eIF2α bands were calculated. Standard deviations from the two independent experiments are shown, and significant differences, as calculated using a t-test and as compared to the vector controls (p < 0.05), are shown. n. s. = non significant. Click here for file
