# Supplementary figures and images for: Characterization of a ranavirus inhibitor of the antiviral protein kinase PKR
Source: BMC Microbiol. 2011 Mar 18;11:56. doi: 10.1186/1471-2180-11-56 (PMC3068933; doi:10.1186/1471-2180-11-56)

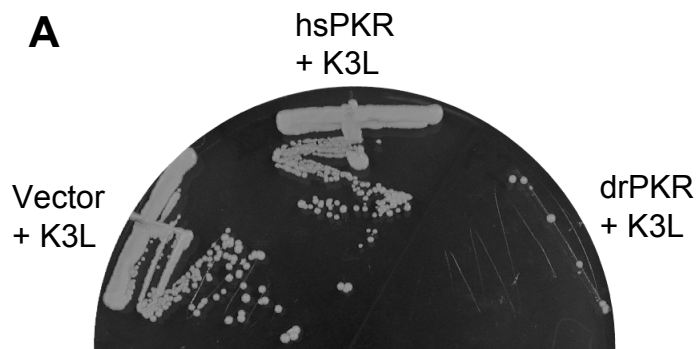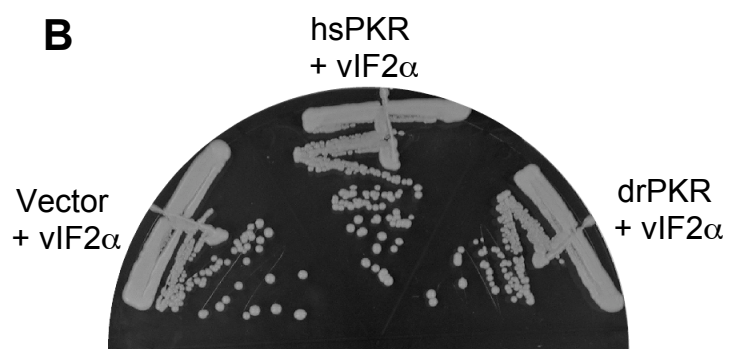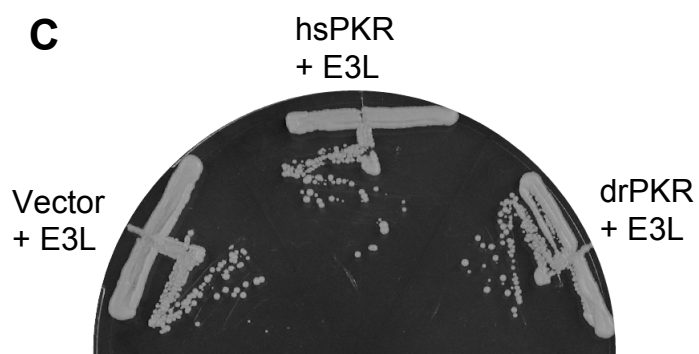

**Additional file 1**

Supplement: Additional file 1 — Figure S1 Comparison of colony sizes of PKR-expressing and control stains expressing K3L, vIF2α or E3L. Plasmids expressing VACV K3L (A, pC140), RCV-Z vIF2α (B, pC3853), or VACV E3L (C, p2245) under the control of a yeast GAL-CYC1 hybrid promoter were introduced into isogenic yeast strains having either an empty vector (J673), a GAL-CYC1-human PKR construct (hsPKR, J983), or a GAL-CYC1-zebrafish PKR construct (drPKR, J944) integrated at the LEU2 locus. The indicated transformants were streaked on SC-Gal medium where expression of both PKR and the viral proteins was induced, and incubated at 30°C for 4 days. Results shown are representative of 4 independent transformants for each plasmid. [file 1471-2180-11-56-S1.PDF]

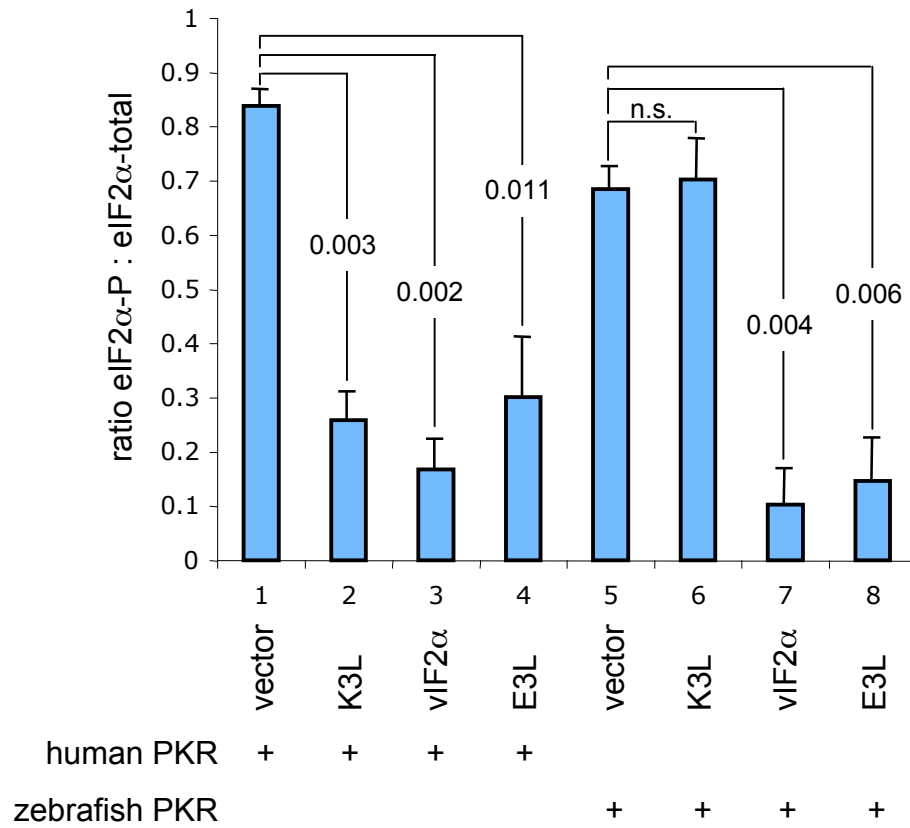

## Additional file 2

Supplement: Additional file 2 — Figure S2 Relative PKR-induced eIF2α phosphorylation levels after expression of vIF2α, K3L or E3L. Using data from Figure 4D and an independent experiment, the band intensities of phosphorylated and total eIF2α obtained from Western blots of TCA extracts of yeast cells expressing either human or zebrafish PKR and transformed with an empty vector or plasmids expressing K3L, vIF2α or E3L, as indicated, were measured using ImageJ. The ratios of phosphorylated and total eIF2α bands were calculated. Standard deviations from the two independent experiments are shown, and significant differences, as calculated using a t-test and as compared to the vector controls (p < 0.05), are shown. n. s. = non significant. [file 1471-2180-11-56-S2.PDF]
